# Anti-type II collagen antibodies are associated with early radiographic destruction in rheumatoid arthritis

**DOI:** 10.1186/ar3825

**Published:** 2012-05-01

**Authors:** Mohammed Mullazehi, Marius C Wick, Lars Klareskog, Ronald van Vollenhoven, Johan Rönnelid

**Affiliations:** 1 Clinical Immunology, Department of Immunology, Genetics and Pathology, Rudbeck Laboratory C5, Uppsala University, Uppsala, SE-75185, Sweden; 2 Department of Medicine, Rheumatology Unit, Building D2:01, Karolinska Institutet, SE-17176, Stockholm, Sweden; 3 Department of Radiology, Innsbruck Medical University, Anichstrasse 35, Innsbruck, A-6020, Austria

## Abstract

**Introduction:**

We have previously reported that high levels of antibodies specific for native human type II collagen (anti-CII) at the time of RA diagnosis were associated with concurrent but not later signs of inflammation. This was associated with CII/anti-CII immune complex (IC)-induced production of pro-inflammatory cytokines *in vitro*. In contrast, anti-cyclic citrullinated peptide antibodies (anti-CCP) were associated both with late inflammation and late radiological destruction in the same RA cohort. We therefore hypothesized that anti-CII are also associated with early erosions.

**Methods:**

Two-hundred-and-fifty-six patients from an early RA cohort were included. Baseline levels of anti-CII, anti-CCP and anti-mutated citrullinated vimentin were analyzed with ELISA, and rheumatoid factor levels were determined by nephelometry. Radiographs of hands and feet at baseline, after one and after two years were quantified using the 32-joints Larsen erosion score.

**Results:**

Levels of anti-CII were bimodally distributed in the RA cohort, with a small (3.1%, 8/256) group of very high outliers with a median level 87 times higher than the median for the healthy control group. Using a cut-off discriminating the outlier group that was associated with anti-CII IC-induced production of proinflammatory cytokines *in vitro*, baseline anti-CII antibodies were significantly (p = 0.0486) associated with increased radiographic damage at the time of diagnosis. Anti-CII-positive patient had also significantly increased HAQ score (p = 0.0303), CRP (p = 0.0026) and ESR (p = 0.0396) at the time of diagnosis but not during follow-up. The median age among anti-CII-positive subjects was 12 years higher than among the anti-CII-negative patients.

**Conclusion:**

In contrary to anti-CCP, anti-CII-positive patients with RA have increased joint destruction and HAQ score at baseline. Anti-CII thus characterizes an early inflammatory/destructive phenotype, in contrast to the late appearance of an inflammatory/destructive phenotype in anti-CCP positive RA patients. The anti-CII phenotype might account for part of the elderly acute onset RA phenotype with rather good prognosis.

## Introduction

A vast majority of patients with rheumatoid arthritis (RA) experience pain, functional deterioration, rigidity and work disability due to atrophy and irreversible joint destruction if not treated efficiently and early. Several different autoantibodies such as rheumatoid factor (RF) [1] and antibodies against citrullinated proteins/peptides (ACPAs), like anti-cyclic citrullinated peptide antibodies (anti-CCP) [2,3] and antibodies against modified citrullinated vimentin (anti-MCV) [4] that have been identified in the serum of patients with RA have a negative prognostic impact on future joint destruction. In earlier studies of a Swedish RA cohort investigated before the systematic introduction of biological agents, we have demonstrated that RF, anti-CCP and anti-MCV detected in serum from patients with RA were associated with late inflammation and late increased rate of radiographic damage [5,6]. In a recently published study we discovered that high levels of anti-native human collagen type II (anti-CII) antibodies in the same group of patients with RA were, in contrast, associated with laboratory measures of inflammation at disease onset [7], which can be explained by pro-inflammatory cytokine induction driven by surface-bound immune complexes (IC) containing anti-CII [8]. We therefore hypothesized that anti-CII antibodies were also associated with early joint destruction in this group of patients with RA.

To address this question, we performed the present study in which we focused on joint destruction in a prospective early RA cohort (*n *= 256), utilizing radiological data from multiple occasions, with parallel investigations of RF, anti-CCP, anti-MCV and anti-CII antibody serum levels.

## Materials and methods

### Patients

In total, 256 patients from a cohort with early RA (< 12 months of disease duration at the time of diagnosis) were included between January 1995 and October 2000. All patients fulfilled the 1987 American College of Rheumatology classification criteria for RA [9]. Sera were obtained at the time of diagnosis and thereafter stored at -70°C and used for the various autoantibody analyses on different occasions. All patients had been given informed consent and the study was approved by the ethics committees at Uppsala University and Karolinska Institutet, respectively.

## Materials and methods

Results about the prognostic impact of anti-CCP [6], anti-MCV [5] and anti-CII on acute inflammation [7], based on a somewhat different patient selection, have been published previously. The 256 patients included in this present analysis represent individuals for whom complete data for RF, anti-CCP, anti-CII and consecutive radiographs were available. Anti-MCV levels were analyzed at a later time point than the other analyses, when 2 out of 256 baseline serum samples were no longer available.

For the anti-CII ELISA that was performed as previously described [7], Maxisorb ELISA plates (Nunc, Roskilde, Denmark) were coated with human native CII (ELISA grade, Chondrex, Redmond, Washington DC, USA, diluted to 2.5 μg/ml in ice-cold PBS immediately prior to coating. Blocking was done with PBS with 1% ELISA grade bovine serum albumin. Serum samples were diluted at 1:100, and antibodies were detected with a F(ab')2 fragmented antibody against human gamma chain that had been pre-adsorbed against bovine proteins (Jackson, Cambridgeshire, UK). Internal controls were investigated together with patient samples on each occasion. The intra-assay coefficient of variation for the internal control close to the cutoff value was 15%.

Radiographs were scored blinded to treatment, in pairs (hands and feet), and in chronological sequence, by an experienced investigator (MCW) using the Larsen method [10]. In each case, 32 joints were scored. The original Larsen score [10] was modified slightly by excluding grade 1 [11], so that the scale became 0 to 4, as described previously [6]. Thus, the maximum possible score was 160. Larsen scores were obtained at baseline, and after one and two years. The change in Larsen score (Δ Larsen score) was calculated by subtracting the baseline score values from the respective annual scores.

### Statistical analysis

**Figure 1:**
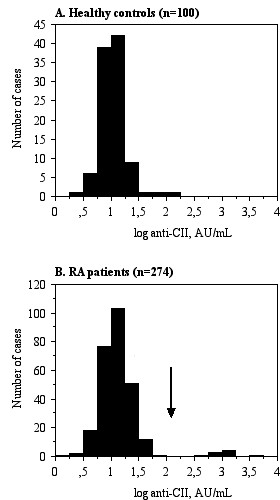
**Distribution of anti-native human collagen type II (anti-CII) antibodies**. **A**) the distribution of anti-CII antibodies among 100 healthy blood donors is shown; **B**) the distribution of anti-CII antibodies among 274 patients with RA [7]; radiological data were available for 256 of these patients. The vertical arrow represents the cutoff value separating the majority of patients with rheumatoid arthritis (RA) and the discrete group with very high anti-CII levels associated with cytokine production in our *in vitro *immune complex assay [7,8].

Non-parametric tests were employed due to the bimodal appearance of anti-CII antibodies. The Mann-Whitney U test was used for the comparison of Larsen score between different groups. *P *values less than 0.05 were considered significant. The cutoff value was placed between anti-CII levels supporting or not supporting IC-induced cytokine production *in vitro *(200 AU/ml) as described previously [7,8]. As can be seen from Figure 1, this cutoff also delimited a patient group with high discrete outlier values.

## Results

Anti-CII-positive patients with RA had higher levels of C-reactive protein (CRP) (*P *= 0.0026) and erythrocyte sedimentation rate (ESR) (*P *= 0.0396) and displayed a higher health assessment questionnaire (HAQ) score (*P *= 0.0303) at the time of diagnosis, compared to anti-CII-negative patients. There was also a trend for higher 28-joint disease activity score (DAS28) among anti-CII-positive patients (*P *= 0.08). The median age at diagnosis was 12 years higher among anti-CII-positive patients (68 vs. 56 years, *P *< 0.05). Baseline characteristics are summarized in Table 1. The increased measures for CRP, ESR and HAQ scores among anti-CII-positive patients were evident only at the time of diagnosis, and not at any other time point between 3 months to 5 years after diagnosis (data not shown).

**Table 1:** Baseline characteristics of the 256 patients with rheumatoid arthritis (RA)

	All RA patients (*n *= 256)	Anti-CII-negative RA patients (*n *= 248)	Anti-CII positive (> 200 AU/ml) RA patients (*n *= 8)	*P *Mann Whitney/chi square
Age at inclusion (years)	56	56	68	NS (0.17)
Female (%: number/total number)	71.5 (183/256)	71.4 (177/248)	75.0 (6/8)	NS (0.82)
Disease duration at inclusion (months)	5.0	5.0	4.5	NS (0.32)
RF-positive, (%; number positive/total number)	63.3 (162/256)	62.9 (158/248)	50.0 (4/8)	NS (0.43)
Anti-CCP2- positive, (%;number positive/total number)	57.8 (148/256)	58.5 (145/248)	37.5 (3/8)	NS (0.24)
Anti-MCV- positive (2 patients missing)	70.9 (180/254)	71.1 (175/246)	62.5 (5/8)	NS (0.60)
CRP (mg/l)	14	14	37	0.0026
ESR (mm/h)	22.0	21.5	40.5	0.0396
Physician's assessment of disease activity (0-4)	2	2	2	NS (0.28)
Number of swollen joints	9.0	9.0	8.5	NS (0.82)
Number of tender joints	7	7	8	NS (0.46)
DAS28	5.005	4.990	5.760	NS (0.08)
Global VAS	45	45	40	NS (0.93)
Pain VAS	45	45	35	NS (0.39)
HAQ	0.880	0.880	1.500	0.0303
Patients starting DMARD therapy at baseline (%)	83.6 (214/256)	83.5 (207/248)	87.5 (7/8)	NS (0.76)

Results are presented as medians and percentages (ratios). Differences between anti-native human collagen type II (anti-CII)-negative and anti-CII-positive patients were analyzed using the Mann-Whitney U test; differences between proportions were analyzed using the Chi square test or Fisher's exact test as appropriate. Anti-CCP, anti-cyclic citrullinated peptide antibodies; Anti-MCV, antibodies against modified citrullinated vimentin; CRP, C-reactive protein; ESR, erythrocyte sedimentation rate; DAS28, 28-joint disease activity score; VAS, visual analogue scale; HAQ, health assessment questionnaire; DMARD, disease-modifying anti-rheumatic drug; NS, not significant.

Comparisons for median Larsen score and changes in median Larsen scores during the first 2 years after diagnosis are presented for the 256 patients with early RA (Table 2). Radiographic scores did not differ between RF-positive and RF-negative patients at any time point. However, analysis of the change in Larsen score revealed increased rates of radiographic damage in RF-positive compared to RF-negative patients between baseline and 2 years (*P *= 0.0354), and especially between 1 year and 2 years (*P *= 0.0009) (Table 2). Similar data, but with stronger statistical significance, were also recorded for anti-CCP and anti-MCV antibodies [5,6].

**Table 2:** Association between baseline autoantibody status and radiological destruction

	RF+ (*n *= 162) vs RF- (*n *= 94)	Anti-CCP + (*n *= 148) vs anti-CCP- (*n *= 108)	Anti-MCV+ (*n *= 180) vs anti-MCV- (*n *= 74)	Anti-CII + (> 200 AU/mL; *n *= 8) versus anti-CII- (*n *= 248)
Larsen score baseline	4.000 versus 5.000, *P *= NS (0.13)	4.000 versus 5.000, *P *= NS (0.16)	4.375 versus 5.000, *P *= NS (0.21)	13.750 versus 4.375, *P *= 0.0486*
Larsen score 1 year	10,500 versus10.500, *P *= NS (0.65)	10.500 versus 10,750, *P *= NS (0.66)	11.000 versus 10.000, *P *= NS (0.99)	21.500 versus 10.500, *P *= NS (0.0799)
Larsen score 2 years	14.000 versus 13.250, *P *= NS (0.69)	14.000 versus 13.000, *P *= NS (0.57)	14.375 versus 10,250, *P *= NS (0.24)	24.000 versus 13.500, *P *= NS (0.19)
ΔLarsen score 1 yr -baseline	4.500 versus 4.250, *P *= NS (0.32)	4.500 versus 4.250, *P *= NS (0.29)	4.500 versus 3.750, *P *= NS (0.19)	7.375 versus 4.250, *P *= NS (0.27)
ΔLarsen score 2 yrs -baseline	7.250 versus 6.250, *P *= 0.0354*	7.500 versus 6.250 *P *= 0.0102*	7.750 versus 5.250, *P *= 0.0028*	9.250 versus 6.500, *P *= NS (0.50)
ΔLarsen score 2 yrs -1 yr	2.500 versus 1.250, *P *= 0.0009*	2.750 versus 1.000, p < 0.0001*	2.750 versus 1.000, p < 0.0001*	3.000 versus 2,.250, *P *= NS (0.48)

Differences in median Larsen score between patients with and without different autoantibodies and changes in median Larsen score are given for 256 patients with early rheumatoid arthritis. Anti-MCV data were missing for two patients. ΔLarsen score, difference in Larsen score; RF, rheumatoid factor; Anti-CCP, anti-cyclic citrullinated peptide antibodies; Anti-MCV, antibodies against modified citrullinated vimentin; Anti-CII, anti-native human collagen type II. *P < 0.05.

**Figure 2:**
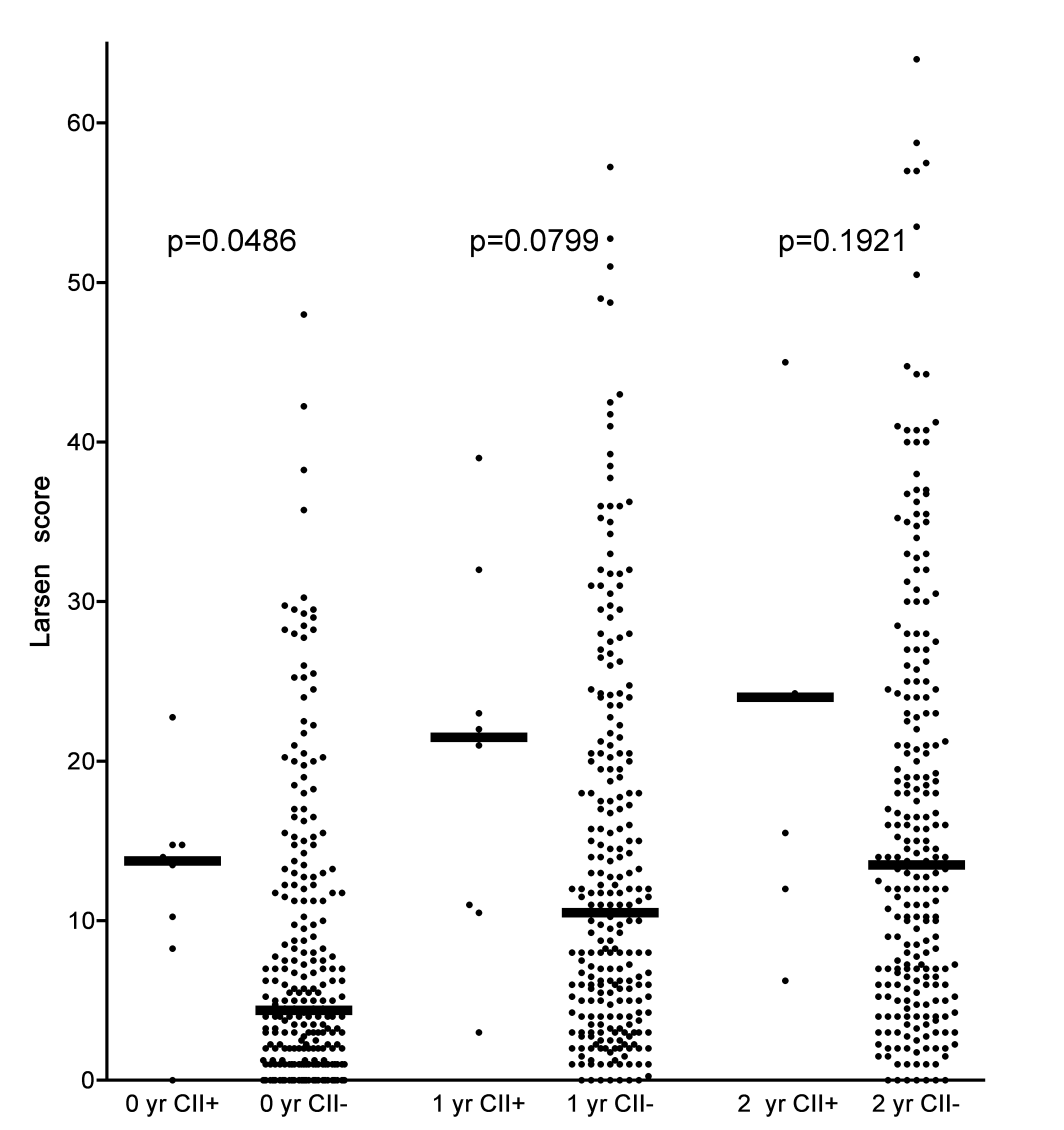
**Radiological destruction in 256 patients with rheumatoid arthritis (RA) at the time of diagnosis, and after one and two years**. Patients are divided into those with high levels of anti-native human collagen type II (anti-CII) antibodies (*n *= 8, arrow in Figure **1B**) and those with lower levels (*n *= 248). Horizontal bars indicate median levels. One patient in the anti-CII group was not investigated at two years.

Whereas anti-CII levels among 100 healthy controls (the same group as used to define the reference range in [7]) showed a normal distribution (Figure 1A), anti-CII levels were bimodally distributed among the patients with RA (Figure 1B). The outlier group was clearly separated from the remaining patients showing a distribution quite similar to the control group, as there were no patients showing anti-CII levels between 57 AU/mL on one hand and the outlier groups with anti-CII levels between 471 and 3520 AU/mL on the other (Figure 1B). The cutoff point used (> 200 AU/mL) was based on this bimodal distribution and our earlier findings that higher anti-CII levels are associated with the functional effects of anti-CII-containing IC formed *in vitro *[7,8]. High anti-CII levels were significantly (*P *= 0.0486) associated with increased Larsen score at baseline (Table 2). A sizeable but non-significant difference between the median anti-CII levels was sustained at all investigated time points (Table 2, Figure 2). There were no differences in changes in Larsen scores between patients with and without anti-CII antibodies (Δ Larsen score; Table 2).

## Discussion

As has been shown earlier, autoantibodies traditionally associated with RA, such as RF [5], anti-CCP [6] and anti-MCV [5], were associated with late increased rates of radiographic destruction in the early RA cohort reported here. In this paper, we have demonstrated that patients with increased levels of anti-CII autoantibodies at the time of diagnosis have more radiographic destructions measured by median Larsen scores at baseline. This is accompanied by a significantly higher HAQ score among anti-CII-positive patients at the time of diagnosis, but not at any later time point.

We can thus distinguish two different phenotypes of patient with RA, *viz*. a group of patients with antibodies traditionally characterized by late inflammation and late increased radiographic destruction, and another group of patients who are positive for anti-CII antibodies, which is characterized by early inflammation [7] and early radiographic destruction.

The Larsen and the Sharp-van der Heide scoring systems are highly correlated [12,13], especially when omitting Larsen grade 1 (soft tissue swelling and periarticular osteoporosis), as was advocated by Arvi Larsen in the modified Larsen scoring system [11]. It is therefore plausible that raised anti-CII levels are associated with breakdown of CII. Such an association might occur either due to a direct effect of anti-CII on cartilage as has been claimed by others [14], or indirectly via induction of pro-inflammatory cytokines by macrophages in the pannus tissue [7,8].

The anti-CII cutoff used in the present study (200 U/mL) was based on the almost dichotomous distribution of anti-CII antibodies among patients with RA shown in Figure 1B, where anti-CII-positive and -negative subjects were clearly separated. Another argument for the use of this cutoff is that the dichotomous distribution of anti-CII among patients with RA clearly distinguished patients whose anti-CII levels permitted IC-induced cytokine induction from those with autoantibody levels too low to induce IL-1β or tumor necrosis factor (TNF) [8]. All anti-CII-negative patients had levels below 60 AU/mL, whereas the anti-CII-positive patients had levels between 471 and 3,520 AU/mL, thus clearly within the range of cytokine induction by the corresponding surface-bound IC [8].

Another major difference between ACPA and anti-CII concerns the kinetics of appearance. Whereas ACPA and RF levels slowly increase years before diagnosis [15,16] and thereafter remain rather stable for years [3,6], anti-CII levels seem to be highest around the time of the beginning of clinical disease. Elevated anti-CII levels do not precede clinical onset of RA [17], and after peaking around the time of diagnosis, levels drop during the first year, according to our own [7] and other [18] investigations. This short window of appearance of anti-CII in early RA, together with our earlier studies linking anti-CII to cytokine-driven inflammation using both functional *in vitro *experiments [8] as well as clinical follow-up studies [7], imply that the long-term effect of initially raised anti-CII levels might be limited, and that the major impact is around the time of disease onset. Initial appearance of anti-CII might in fact be prognostically advantageous. As initially raised anti-CII levels are associated both with raised CRP and ESR [7] and with augmented joint destruction around the time of diagnosis, and as these differences are no longer evident after one year, patients with RA and an early appearance of anti-CII might erroneously give an impression of a more severe prognosis than is actually the case. The general belief among rheumatologists is that a certain subgroup of patients with acute disease onset eventually have a rather good prognosis and need only rather limited treatment. This has been formally proven, as a recent study showed that both acute onset of RA and short symptom duration before inclusion in the study are associated with drug-free remission [19]. We speculate that at least some of these patients belong to the initially anti-CII-positive RA subgroup. This hypothesis should be investigated in other follow-up cohorts.

In this study, early RA was defined as < 12 months of symtoms before enrolment. Given the fact that anti-CII levels often drop sharply within the first months [7], we cannot rule out that some initially anti-CII-positive patients might have dropped to levels below the cutoff before diagnosis of RA.

The more active onset of destructive joint disease in anti-CII-positive RA patients might also initiate an earlier health care contact by patients after the first apearance of joint symtoms. This was also the case in our earlier larger study, where anti-CII-positive patients had a significantly shorter duration of joint symtoms before diagnosis of RA [7]. Acute onset RA is common among older individuals [20], and the median age among our anti-CII-positive patients was 12 years higher than among anti-CII-negative patients (Table 1).

One earlier study has also implicated an association between anti-CII and simultaneously occurring inflammation in RA. In the study by Kim et al [21], patients with anti-CII had, like our patients [7], higher levels of CRP and ESR than antibody-negative patients with RA, and also had higher levels of TNF and IL-6. We presume that both these cytokines have been produced by macrophages stimulated with anti-CII-containing IC. In our studies we have shown that anti-CII containing IC induces the production of TNF, IL-1β and IL8 from monocytes/macrophages [7,8]. In these experiments we chose not to study IL-6, due obscuring of the *in vitro *signal by high basal IL-6 production. In other systems we have, however, found patient-derived IC to induce IL-6 in our *in vitro *models [22-24]. In contrast to our present investigation, the study by Kim et al could not show increased radiologic progression among anti-CII-positive patients with RA, a finding that can be explained by the fact that the patients had long-standing disease with a mean duration of 75.8 months [21]. In our study the significant difference in Larsen score between anti-CII-positive and -negative patients was only evident at the time of diagnosis, and was no longer visible after one year. Even if our patients were all included within 12 months of first symtoms, the mean interval between the first symtoms and diagnosis of RA was 5.8 months [7]. It is therefore possible that we would have found bigger differences between the groups if patients with shorter duration of joint symtoms had been included.

In conclusion, anti-CII antibodies are associated with early radiological destruction at the time of diagnosis, while autoantibodies traditionally associated with RA, such as RF, anti-CCP and anti-MCV are later associated with increased rates of radiographic destruction. This finding is in agreement with our earlier discovery that the same groups of antibodies are inversely associated with early [7] and late [5,6] signs of inflammation in the same patient cohort. It is intriguing to note that the phenotypically opposite ACPA and anti-CII phenotypes, at least in this patient cohort, are also statistically inversely related (*P *= 0.04) as previously described [7]. We speculate that CII antibodies might stimulate macrophages and fibroblasts within the synovium to produce the metalloproteinases that are responsible for being the first enzymes to cleave the interstitial collagens, resulting in bone and cartilage destruction. We are currently performing studies to address this issue.

## Conclusions

The occurrence of high levels of anti-CII antibodies characterizes a small group of patients with RA who have an elevated degree of joint destruction and elevated HAQ score at the time of diagnosis. Anti-CII characterizes an early inflammatory/destructive RA phenotype, in contrast to the late inflammatory/destructive phenotype that is associated with ACPA.

## Abbreviations

ACPA: antibodies against citrullinated proteins/peptides; anti-CII: anti-native human collagen type II antibodies; anti-CCP: anti-cyclic citrullinated peptide antibodies; anti-MCV: antibodies against modified citrullinated vimentin; CRP: C-reactive protein; DAS28: 28-joint disease activity score; DMARD: disease-modifying anti-rheumatic drug; ESR: erythrocyte sedimentation rate; HAQ: health assessment questionnaire: IC: immune complex; PBS: phosphate buffer saline: RA: rheumatoid arthritis; RF: rheumatoid factor; TNF: tumor necrosis factor.

## Competing interests

The authors declare that they have no competing interests.

## Authors' contributions

MM planned the study, carried out collagen antibody analyses, performed statistical analyses, and drafted the manuscript. MCW performed Larsen scoring and drafted the manuscript. LK took part in the planning discussions and drafted the manuscript. RvV supervised Larsen scoring and drafted the manuscript. JR conceived the study, performed statistical analyses, and drafted the manuscript. All authors read and approved the final manuscript.
